# Prevalence and predictors of intimate partner violence against men in Kisumu slums, Kenya

**DOI:** 10.21203/rs.3.rs-3489793/v1

**Published:** 2023-10-26

**Authors:** Elizabeth Odemba, Edward Frongillo, Sheri Weiser

**Affiliations:** Department of Public Health, Maseno University, P.O Box 811, Kisumu, Kenya; Department of Health Promotion, Education, and Behavior, University of South Carolina.; University of California and San Francisco’s Division of HIV, Infectious Diseases and Global Medicine, University of California and Sans Francisco

**Keywords:** Prevalence, slums, intimate partner violence, community health volunteers

## Abstract

**Background:**

Men in sub-Saharan Africa experience intimate partner violence, with few reporting their cases to the legal authorities or coming out for assistance. Consequently, data on the prevalence and drivers of intimate partner violence in different parts of sub-Saharan Africa are inadequate. Therefore, this study was designed to investigate the prevalence and predictors of intimate partner violence against men in Kisumu slums, Kenya.

**Methods:**

This retrospective cross-sectional study included 398 randomly selected male participants from Kisumu slums, sampled data collected from Community Health Volunteers. We used a multinomial regression analysis to assess determinants and forms of violence.

**Results:**

A total of 398 respondents out of 438 eligible men participated in the survey. The prevalence of intimate partner violence against men was 76.1%. From the multinomial regression, men who were married or living together, compared with never married, were 2.13 times more likely to have experienced physical violence (95% CI = 0.91–4.97, p = 0.080) and 2.41 times more likely to have experienced economic violence (95% CI = 1.20–4.84, p = 0.013). Compared to never married, men who divorced or separated were 5.42 times more likely to have experienced sexual violence (95% CI = 0.97–30.37, p = 0.055). Men who had primary education or less were 2.39 times more likely to have experienced sexual violence (95% CI = 1.02–5.61, p = 0.045). Men who were Muslim, compared with Protestants, were 2.37 times more likely to have experienced psychological or emotional violence (95% CI = 0.87–6.37, p = 0.086).

**Conclusions:**

Sexual, physical, and emotional violence is common among men in Kisumu slums, and the prevalence differs by age, marital status, education, and religion. Safe spaces should be created that will enable men of diverse socio-demographic characteristics to share their experiences of violence by intimate partners. Policies, including education to increasing awareness of this issue, should be enacted to protect men from intimate partner violence.

## Background

Intimate partner violence (IPV) is any form of harm or aggression on a partner that could cause sexual, psychological, economic or physical violence [1]. Whereas there is a robust body of literature investigating male-perpetrated IPV [2], there is a dearth of literature examining IPV perpetrated by women on men [3]. Yet the World Health Organization has viewed IPV against men as a significant public health issue since 2013. Globally, the prevalence of lifetime IPV has been reported to be between 10% and 52% of men in marriage [2]. Estimates from National Family Violence Surveys in the United States show that within a given year, at least 12% of men are the targets of physical aggression from their female partners, and 4% or over 2.5 million men sustain severe violence [3].

Few studies have investigated female domestic violence towards men in sub-Saharan Africa, which is not surprising because, in this part of the world, gender relations are unequal, with patriarchy and male-dominated cultures as the norm. Therefore, the notion of female domestic violence against men would be viewed with scepticism [4]. As a result of difficulties collecting these data in sub-Saharan Africa, the reported prevalence of IPV perpetrated by females against men varies widely in studies ranging from 9–71% [5, 6]. The Zambia Demographic and Health Survey indicates that 9% of women reported having initiated physical violence against their husbands, and 5% had done so in the past 12 months [7]. The Kenya Demographic and Health Survey has revealed that 44% of men aged 15–49 years reported to have suffered some form of violence during their lifetime from their female partners [8].

Most literature suggests that women only engage in violence in self-defense, fear or retribution for real or perceived wrongdoing on the part of men [9]. Self-defense is often listed as a motivation for committing violence. Some women’s violence occurs in the context of fear of assault from their partners and the need to protect themselves from physical harm [10]. In addition, a vital link has been found between male alcoholism and the occurrence of IPV in many countries. This link may be because excessive drinking by male partners can exacerbate financial difficulties and childcare problems and can contribute to their infidelity and irresponsible behaviour. Alcohol use by male partners increases the occurrence and severity of female-perpetrated IPV [11].

According to the most recent Kenya Demographic and Health Survey 2014 data, 56.3% of men in the Nyanza region have experienced physical violence and 13.4% sexual violence, making the region particularly vulnerable to IPV towards men as compared to other counties in Kenya [8]. Kisumu has the highest number of slums in Nyanza, and the prevalence of IPV is particularly high in slums [12]. Few studies have investigated the correlates of IPV against male populations. In addition, a few studies on the topic were conducted before 2014, with limited data on the prevalence and correlates of female-perpetrated IPV to males since then. Addressing this gap is essential since understanding the current correlates of IPV towards men can help devise interventions, particularly in regions like Kisumu, where the prevalence is high.

The aim of this study was to determine the prevalence and predictors of IPV against men using a random sample of men living in Kisumu slums.

## Methods

This cross-sectional study involved semi-structured questionnaires in which the respondents received the survey via research assistants. The survey was conducted in July 2019 in Kisumu slums. We selected a random sample of 398 respondents among the 81,882 men living in slums aged 18–54 years [13]. We used two stages of probability sampling in which stage one involved probability proportional to size that was used to determine the number of slums to include in the study out of the eight slums in Kisumu municipality. Seven out of eight slums were thus obtained. Stage two involved simple random sampling that helped obtain individual respondents from the slums to be surveyed. The sampling frame was from the community health volunteer registers.

Piloting was done in Kanyakwar-Obunga, a slum excluded from the study. We used a content validity technique to develop the questionnaires where public health professionals with experience in handling domestic violence assessed the relevance of the content used in the questionnaire developed.

Six trained research assistants conducted the surveys where data were collected on socio-demographic variables and forms of IPV in men within the seven sampled slums. All researchers were trained on research ethics and how to handle sensitive questions. We obtained written informed consent from all participants, and the study received ethical approval from Maseno University Scientific Ethics Review Committee, proposal reference number MSU/DRPI/MUSERC/00715/19.

The primary outcome was IPV, as assessed using the questionnaire with data validation done in Kisumu, Kenya. IPV was seen as a form of violence inflicted on a man as physical, sexual, emotional, or economical. Physical violence entailed harm to a man using objects like a knife or a club. Sexual violence was defined as a forceful engagement in sexual activity by the intimate partner without the man’s consent. Emotional violence entailed insults or the use of derogatory words towards a man from the intimate partner. Economic violence was defined as the denial of access to his money accounts or assets or money they shared.

Statistical analysis was carried out using Stata version 18.0 (StataCorp, College Station, TX). Descriptive statistics, including the mean, standard deviation (SD), and percentages, were estimated. The prevalence of IPV was determined based on experience with IPV. We used multinomial regression to investigate the predictors of IPV with a sample of 394 participants who reported whether they had ever experienced IPV and the forms of violence. The dependent variable was constructed as five nominal categories: no IPV, physical violence, sexual violence, emotional violence, and economic violence. The no IPV category was used as the reference. The predictor variables were marital status (never married as referent compared to married/living together and divorced/separated/widowed); education (secondary or higher as referent compared to no or primary education); religion (Protestant as referent compared to Roman Catholic, Muslim, and no religion) and employment status (employed as referent). Multinomial regression produced relative risk ratios (RRR) and not odds ratios [14].

## Results

A total of 398 respondents out of 438 eligible men (91%) participated in the survey. The prevalence of IPV against men was 76.1% (Table 1). Out of the five forms of violence, emotional violence emerged as the most common form at 47.5%, economic at 23.8%, and sexual violence at 16.5%. The least common form of violence experienced by men in Kisumu slums was physical violence at 12.2%. There was a 1% non-response to the survey on IPV.

The mean age of men in the study who never experienced intimate partner violence was 32.3 ± 7.62 years (Table 2). Men who experienced physical violence were of mean age 32.8 ± 8.00 years, while those who experienced sexual violence were of mean age 34.2 ± 9.49 years. Emotional and economic violence were relatively equal at 31.8 ± 8.26 and 31.3 ± 7.80 years, respectively.

IPV was less common among those who were never married at 56.0%. Married men or those living together with their intimate partners encountered economic and physical violence at 58.3% and 56.8%, respectively, as compared to 37.5% and 37.8% on economic and physical violence of their never-married counterparts.

Overall, men with a higher level of education experienced more intimate partner violence than men with only a primary education or no education. Men with higher education levels experienced physical violence at 83.8%, followed by psychological and economic violence, with both at 79.2%. In contrast, only 16.2% of men with lower education reported physical violence.

Men who never experienced IPV were more than two-thirds (74.7%) Protestants. Men experiencing physical and economic violence were 81.1% and 81.9% Protestant, respectively.

A significant number (60.4%) of men experiencing psychological violence were employed, followed by sexual violence at 54.0%. Most (55.6%) of men experiencing economic violence were unemployed.

From the multinomial regression, men who were married or living together, compared with never married, were 2.13 times more likely to have experienced physical violence (95% CI = 0.91–4.97, p = 0.080) and 2.41 times more likely to have experienced economic violence (95% CI = 1.20–4.84, p = 0.013) (Table 3). Compared to never-married, divorced or separated men were 5.42 times more likely to have experienced sexual violence (95% CI = 0.97–30.37, p = 0.055). Men who had none or primary education were 2.39 times more likely to have experienced sexual violence (95% CI = 1.02–5.61, p = 0.045). Men who were Muslim, compared with Protestants, were 2.37 times more likely to have experienced psychological or emotional violence (95% CI = 0.87–6.37, p = 0.086).

## Discussion

The prevalence of domestic violence against men was high at 76.1% in the Nyanza region of Kenya. This is a major public health concern since IPV has many adverse health effects, including depression, suicidality, withdrawal from peers and family, anxiety, post-traumatic stress disorder, cardiovascular disease and premature mortality [15]. The prevalence of IPV in this study is higher than that reported in many studies in different parts of the world. A study published in the USA by the National Library of Medicine on the prevalence and risk factors of domestic violence against men determined domestic physical violence prevalence rates of 3.4–20.3% [16]. The prevalence of domestic violence against men in Mumbai slums, India, was 36.9% [17]. The prevalence of IPV against men was reported to be 55.4% in a cross-sectional study conducted in Nigeria among 410 men aged 20 to > 60 years [18].

In our study, men who were married or living together with their female intimate partners were more likely to experience domestic violence than those men who were never married or divorced. It is unclear whether this is because unmarried and divorced men have lower contact with female sexual partners or there are other underlying reasons. The result from this study is contrary to that of other studies about the role of marriage and men’s health, which have found that, in general, marriage is beneficial to men’s health because of the social support and companionship conferred by marriage. For example, a study conducted at Harvard School of Medicine in 2019 on marriage and men’s health found that married men or men with marital partners live healthier and longer compared to never-married men or whose marriages ended in divorce or widowhood [19]. This is also supported by a study on family relationships, which reported that marriage helps reduce stress and depression. It also helps survive heart attacks and lowers the chances of developing cancer and dementia [20].

Contrary to expectations and to the existing literature, we found that more educated men in this study experienced more IPV than men with lower education levels. Previous research has shown that education has a largely positive effect on reducing gender-based violence [21], with women who had the highest education levels being at least likely to perpetrate violence towards men [21]. In many studies, women have been found to engage in intimate partner violence because of reactive motives (responding to a perceived threat, such as defending oneself when attacked) versus proactive (aggression that is initiated to dominate, control, threaten, or bully someone else) [22]. Women’s violence towards their educated partners could result from defense [23].

Overall, psychological violence in this study was rampant, cutting across all ages, marital status, education, and employment. This result is consistent with several studies involving violence against men by their intimate partners. For example, earlier results from the Kenya Demographic and Health Survey in 2014 showed that 20.9% of men experienced psychological violence, a percentage that was higher than the other forms of violence reported. This type of violence has important mental health consequences. A study reported that abused men were more likely to experience psychosomatic symptoms, stress and depression than non-abused men [24]. This was backed up by another study showing that abused men are at risk of emotional hurt, fear, depression, stress, psychological distress and psychosomatic symptoms [25].

This study used a cross-sectional design, which limits causal inference or an understanding of changes in IPV over time. The IPV measures were self-reported, and there may have been over or under-reporting of cases to the authorities due to fears of breach of confidentiality. In protection of confidentiality, no names were recorded on the surveys, and all records were kept secure using password-protected files.

## Conclusion

IPV against men is a prevalent public health concern with significant consequences for health and well-being. Overall, psychological violence was most common across all ages, marital status, educational levels, and employment types. Since IPV against men is associated with depression and poor mental health, which in turn contributes to worse chronic disease, there is a need to increase sensitization within the community on the importance of health-seeking for male IPV. This education should start in schools so that boys are prepared to seek help and respond. Safe spaces should be created that will enable men of diverse socio-demographic characteristics to share their experiences of violence by intimate partners.

## Figures and Tables

**Table 1:** Prevalence and forms of male IPV

Variable	Count (%)

**Ever experienced IPV**	
Yes	303 (76.1)
No	91 (22.9)
No response	4 (1.0)

**Total**	
**Forms of violence**	**398 (100.0)**
Physical violence	37 (12.2)
Sexual violence	50 (16.5)
Emotional violence	144 (47.5)
Economic violence	72 (23.8)
**Total**	**303 (100.0)**

The table presents the prevalence of IPV, forms of violence and the counts in percentages.

**Table 2:** Demographic characteristics and forms of violence

Forms of Violence					

	No IPV	Physical violence	Sexual violence	Psychological violence	Economic violence

**Age** mean (SD)	32.3(7.62)	32.8(8.00)	34.2(9.49)	31.8(8.26)	31.3(7.80)

**Marital status**					
Never married	56.0	37.8	46.0	49.3	37.5
Married or living together	41.8	56.8	44.0	44.4	58.3
Divorced/separated/widowed	2.2	5.41	10.0	6.25	58.3

**Education**					
None/ Primary level	15.4	16.2	30.0	20.8	20.8
Secondary +	84.6	83.8	70.0	79.2	79.2

**Religion**					
Roman Catholic	15.4	8.1	16.0	13.9	6.9
Protestant	74.7	81.1	76.0	69.4	81.9
Muslim	6.6	10.8	6.0	13.9	8.3
No religion	3.3	0.0	2.0	2.8	2.8

**Employment**					
Employed	52.6	51.4	54.0	60.4	44.4
Not employed	47.3	48.7	46.0	39.6	55.6

Numbers are percentages except for the age.

The table illustrates the demographic characteristics of the study participants and forms of violence, including no experience of IPV.

**Table 3:** Association between demographic determinants and types of violence

			95.0% C.I	

	RRR	ρ-value	Lower	Upper

	Physical Violence			

**Marital status**				
Never married (ref)	--	--	--	--
Married/ living together	2.129	**0.080**	0.912	4.965
Divorced/separated/widowed	3.267	0.259	0.418	25.522
**Education**				
None/primary	0.937	0.905	0.324	2.714
Secondary+ (ref)	--	--	--	--
**Religion**				
Roman Catholic	0.503	0.311	0.133	1.900
Protestant (ref)	--	--	--	--
Muslim	1.801	0.400	0.457	7.093
No religion	3.13	0.983	--	--
**Employment**				
Not employed	1.190	0.672	0.532	2.662
Employed (ref)	--	--	--	--
Constant	0.264	0.001	0.116	0.599

**Sexual Violence**				

**Marital status**				
Never married (ref)	--	--	--	--
Married/ living together	1.075	0.855	0.497	2.324
Divorced/separated/widowed	5.416	**0.055**	0.966	30.367
**Education**				
None/primary	2.390	**0.045**	1.020	5.607
Secondary+ (ref)	--	--	--	--
**Religion**				
Roman Catholic	1.016	0.974	0.384	2.687
Protestant (ref)	--	--	--	--
Muslim	0.786	0.751	0.178	3.473
No religion	0.672	0.740	0.064	7.049
**Employment**				
Not employed	0.959	0.911	0.461	1.995
Employed (ref)	--	--	--	--
Constant	0.419	0.015	0.208	0.845

**Psychological/ Emotional violence**				

**Marital status**				
Never married (ref)	--	--	--	--
Married/ living together	1.177	0.581	0.660	2.100
Divorced/separated/widowed	3.245	0.145	0.666	15.857
**Education**				
None/primary	1.346	0.415	0.659	2.748
Secondary+ (ref)	--	--	--	--
**Religion**				
Roman Catholic	0.924	0.838	0.432	1.974
Protestant (ref)	--	--	--	--
Muslim	2.374	0.086	0.886	6.366
No religion	1.160	0.852	0.243	5.544
**Employment**				
Not employed	0.698	0.208	0.399	1.222
Employed (ref)	--	--	--	--
Constant	1.443	0.168	0.857	2.428

**Economic violence**				

**Marital status**				
Never married (ref)	--	--	--	--
Married/ living together	2.412	**0.013**	1.204	4.835
Divorced/separated/widowed	2.626	0.310	0.407	16.933
**Education**				
None/primary	1.289	0.547	0.565	2.942
Secondary+ (ref)	--	--	--	--
**Religion**				
Roman Catholic	0.433	0.133	0.145	1.292
Protestant (ref)	--	--	--	--
Muslim	1.367	0.615	0.405	4.605
No religion	1.081	0.935	0.166	7.048
**Employment**				
Not employed	1.623	0.150	0.839	3.138
Employed (ref)	--	--	--	--
Constant	0.391	0.007	0.198	0.774

The table presents the association between demographic determinants and the types of violence, including the RRR, ρ-value and 95% confidence interval.

## Data Availability

The datasets used and/or analysed during the current study are available from the corresponding author on reasonable request.

## List Of Abbreviations

IPV: Intimate Partner Violence
